# Supplementary material for: Clinical predictors of encephalitis in UK adults–A multi-centre prospective observational cohort study
Source: PLoS One. 2023 Aug 23;18(8):e0282645. doi: 10.1371/journal.pone.0282645 (PMC10446234; doi:10.1371/journal.pone.0282645)
Supplement: S1 Table — (DOCX) [file pone.0282645.s001.docx]

## S1 Table. Multivariate logistic regression model with selected variables for autoimmune encephalitis (confirmed, probable and possible) versus other forms of encephalitis

| **Selected variables from the model** | **Parameter**  **estimate** | **Standard error** | **Odds ratio** | **95% CI of OR** | **p-value** |
| --- | --- | --- | --- | --- | --- |
| **Intercept** | -0.53 | 0.30 | 0.59 | 0.33,1.05 | 0.07 |
| **History Fever** | -1.10 | 0.39 | 0.33 | 0.15,0.72 | <0.01 |
| **Abnormal movement** | 1.65 | 0.41 | 5.25 | 2.36,11.72 | <0.01 |
| **CSF WCC ≥20** | -1.16 | 0.38 | 0.31 | 0.15,0.66 | <0.01 |

Multivariate analysis was performed using 19 clinical and laboratory variables at presentation that were significant selected from the univariate analysis. The results were fitted using step Bayesian information criterion using backward selection.
