# Supplementary material for: Clinical predictors of encephalitis in UK adults–A multi-centre prospective observational cohort study
Source: PLoS One. 2023 Aug 23;18(8):e0282645. doi: 10.1371/journal.pone.0282645 (PMC10446234; doi:10.1371/journal.pone.0282645)
Supplement: S2 Table — (DOCX) [file pone.0282645.s002.docx]

S2 Table. Presenting clinical features and outcomes of the autoantibody mediated forms of autoimmune encephalitis

|  |  | Antibody defined encephalitis (26) | | | | |  |
| --- | --- | --- | --- | --- | --- | --- | --- |
|  |  | LGI-1 (1) | NMDAR (9) | GAD (5) | Hu (1) | IgLON5(1) |  |
|  | Age (med. IQR) | 74 | 26 (22, 67) | 29 (22, 30) | 59 | 45 |  |
|  | Female | 1 | 6 (67%) | 3 (60%) | 1 (100%) | 0 (0%) |  |
|  | White | 1 | 9 (100%) | 5 (100%) | 1 (100%) | 1 (100%) |  |
|  | Co-morbidity (Charlson) | 0 | 1 (11%) | 1 (20%) | 1 (100%) | 0 (0%) |  |
|  | Immunocompromise | 0 | 0 | 0 | 0 | 0 |  |
|  | **Symptoms** | | | | | |  |
|  | History of Fever | 0 | 3 (33%) | 1 (20%) | 1 (100%) | 0 |  |
|  | History of Rash | 0 | 1 (11%) | 1 (20%) | 0 | 0 |  |
|  | Flu like symptoms | 0 | 1 (11%) | 0 | 0 | 0 |  |
|  | Seizures | 0 | 6 (67%) | 3 (60%) | 1 (100%) | 0 |  |
|  | Altered personality/ behaviour | 1 | 8 (89%) | 5 (100%) | 0 | 1 (100%) |  |
|  | Agitation | 1 | 5 (56%) | 1 (20%) | 0 | 1 (100%) |  |
|  | Lethargy/ increased sleeping | 0 | 3 (33%) | 3 (60%) | 0 | 0 |  |
|  | Psychosis | 0 | 2 )22%) | 1 (20%) | 0 | 0 |  |
|  | Severe/worst ever headache | 0 | 1 (11%) | 1 (20%) | 0 | 0 |  |
|  | Confusion/disorientation | 1 | 6 (67%) | 1 (20%) | 1 (100%) | 1 (100%) |  |
|  | Language/ speech problems | 1 | 3 (33%) | 3 (60%) | 1 (100%) | 1 (100%) |  |
|  | Memory problem | 1 | 5 (56%) | 2 (40%) | 0 | 1 (100%) |  |
|  | Hallucinations | 0 | 2 (22%) | 0 | 0 | 0 |  |
|  | **Examination findings** | | | | | |  |
|  | Fever | 0 | 2 (22%) | 0 | 1 (100%) | 0 |  |
|  | GCS ≤12 | 0 | 2 (22%) | 0 | 0 | 0 |  |
|  | Focal weakness | 0 | 0 | 0 | 0 | 1 (100%) |  |
|  | Abnormal movements | 0 | 5 (56%) | 3 (60%) | 1 (100%) | 1 (100%) |  |
|  | Rash | - | 2 (22%) | 1 (20%) | 0 | 0 |  |
|  | **Investigation findings** |  |  |  |  |  |  |
|  | Median CSF WCC (IQR) | 5 | 9 (6, 32) | 2 (0, 13) | 17 | 14 |  |
|  | Median blood sodium (IQR) | 136 | 139 (136, 140) | 134 (131, 138) | 135 | 136 |  |
|  | Hyponatraemia | 0 | 2 (22%) | 3 (60%) | 0 | 0 |  |
|  | Abnormal initial CT head | 0 | 0/9 (0%) | 0/3 (0%) | 1/1 (100%) | 0/1 (0%) |  |
|  | Abnormal initial MRI head~ | 1 | 1/9 (11%) | 2/5 (40%) | 1/1 (100%) | 0/1 (0%) |  |
|  | Abnormal EEG | 1 | 8/8 (100%) | 2/4 (50%) | 1/1 (100%) | 1/1 (100%) |  |
|  | **Inpatient progress & outcome** | | | | | |  |
|  | ICU admission | 0 | 6/9 (67%) | 2/5 (40%) | 0 | 0 |  |
|  | Hosp LOS Median (IQR) | 28 | 80 (25, 89) | 110 (41, 160) | 37 | 17 |  |
|  | Mortality admission | 0 | 1 (11%) | 1 (20%) | 0 | 0 |  |
|  | GOS at discharge | 4 | 4 (3, 4) | 3.5 (2.5, 4.25) | 4 | 4 |  |
|  | **Outcomes at 12 months** | | | | | |  |
|  | Mortality at 12 months | 0 | 1 (11%) | 1 (20%) | 0 | 0 |  |
|  | Median GOS at 12 months | 5 | 5 (5, 5) | 3.5 (2.5, 4.25) | Unk | 4 |  |
|  |  | | | | | |  |
