# Supplementary material for: Clinical predictors of encephalitis in UK adults–A multi-centre prospective observational cohort study
Source: PLoS One. 2023 Aug 23;18(8):e0282645. doi: 10.1371/journal.pone.0282645 (PMC10446234; doi:10.1371/journal.pone.0282645)
Supplement: Attachment — Submitted filename: Response to reviewers letter.docx [file pone.0282645.s004.docx]

**Staff Editor	Liverpool Brain Infection Group**

**PLOS ONE	Institute of Infection Veterinary & Ecological Sciences**

**Ronald Ross Building**

**University of Liverpool**

**Dear Mr Tischer	28/6/2022**

**Thank you for reviewing our manuscript. Please find here our responses to the points raised by the editor and reviewer.**

**Point 1 Please ensure that your manuscript meets PLOS ONE's style requirements, including those for file naming.**

**We have reviewed the manuscript in line with the guidance on format and affiliations and have corrected these.**

Point 2. We note that the grant information you provided in the ‘Funding Information’ and ‘Financial Disclosure’ sections do not match.

**Thank you for highlighting this point and we apologise for the inconsistency in submitting this. We have ensured that the grant information in the funding information section and financial disclosure sections match.**

When you resubmit, please ensure that you provide the correct grant numbers for the awards you received for your study in the ‘Funding Information’ section.

Point 3. Please amend the manuscript submission data (via Edit Submission) to include author Michael J Griffiths.

**We have added author Michael J Griffiths in the submission data.**

Point 4. One of the noted authors is a group or consortium [insert name of group or team]. In addition to naming the author group, please list the individual authors and affiliations within this group in the acknowledgments section of your manuscript. Please also indicate clearly a lead author for this group along with a contact email address.

**We have checked through the group authorship team details. Each author has been named along with their affiliations in the acknowledgment section and the lead author has been detailed along with their contact email address**

**Response to the reviewers comments in point 5**

**We acknowledge that it is late in getting published as the team have unfortunately been significantly impacted by the global pandemic. In particular, the first author helps run a large supra-regional infectious department covering the Northwest of England and therefore had a large clinical workload at the time when submission was planned.**

**Thank you to the Reviewer for raising the point with regards to additional post-hoc serological studies to identify further autoimmune encephalitis antibodies. We acknowledge that we did not make clear in the manuscript that these additional tests were performed in the group of ‘encephalitis of unknown aetiology’ using the full panel of autoimmune antibodies available. In addition, further serologies for infectious aetiologies was evaluated for those with a appropriate travel history to identify any travel-related infections that may have been missed at the source hospitals. We have edited the revised manuscript to make this all more clear in the methods section. After these key investigations were performed only limited samples remained and more exploratory testing was not possible. Similarly, after clinically indicated investigations, there was insufficient left-over CSF samples to explore the presence of autoimmune antibodies beyond those investigations performed at original recruiting hospitals.**

**We acknowledge that testing for VGKC complex antibodies evolved during the course of the study. Early samples were reported only as VGKC complex positive where serum levels were >100pmol/L. Later in the study laboratories were assessing the presence of antibodies for LGI1 and CASPR 2 proteins by cell-based assays. Of the 10 patients with positive antibodies to VGKC complex (at significant levels and indeed some very high), 5 (50%) also had evaluation for LGI-1 and CASPR 2 antibodies. None of these were positive. The remaining 5 did not have these additional tests performed as insufficient volumes of sample from the period of clinical illness were available. Interestingly, 3 of the patients who were tested and found to be negative for these 2 proteins were fairly young, (18, 23 and 24 years) and in keeping with the paediatric population and their presentation. Of those not tested for these subunits, 3 of 5 (60%) had abnormal movement disorders.**

**At the advice of the Reviewer, we have included the following in the revised manuscript:**

All patients with suspected autoimmune encephalitis had, as a minimum, tests to detect NMDAR, glutamic acid decarboxylase (GAD) and paraneoplastic antibodies. For VGKC-complex antibodies (LGI-1, CASPR-2 and Contactin) diagnostic panels evolved over the course of this observational study. Whenever possible the detection of antibodies directed at specific epitopes of the VGKC-complex are presented.

**We agree with the Reviewer that the study has an intentional selection bias for rapid and subacute presentations by the initial criteria chosen for entry into the study. This was chosen to reflect the majority of presentations of encephalitis faced by treating clinicians to inform early investigatory and management decisions. Although we acknowledge that this may under-represent some of the rarer and more gradual onset cases such as IgLon-5 antibodies. At the advice of the Reviewer this has been included in the limitations section of the discussion:**

Firstly, although ours was one of the largest prospective studies of unselected patients with encephalitis, the size was limited, especially in terms of numbers with specific autoimmune diagnoses and also this study was intentionally biased towards those autoimmune causes that would present acutely or sub-acutely, similarly to most infectious cases in the differential, as seen by general clinicians. Although this study design may have decreased the likelihood of capturing rarer antibody cases (such as IgLon-5) which present with a more gradual and less acute symptoms

**We have added this to the discussion regarding potential areas of bias of the study and also added, as requested, the supplementary material of the clinical features of the autoantibody cases of encephalitis. Though we hasten to add these are small numbers.**

**We agree with the Reviewer that** **the lack of rash being associated with HSV-1 when compared to other types of encephalitis is, in the main part, due to the rash present in cases of encephalitis due to VZV. Whilst rashes are known to be associated with a range of viral infections including enterovirus, Epstein Barr virus, flavivirus group, influenza and adenoviruses, the majority of rashes in this cohort occurred in cases of VZV encephalitis. However, not all cases of VZV encephalitis had a rash present.**

**This has been acknowledged in the revised manuscript:**

The lack of rash being associated with HSV-1 when compared to other types of encephalitis is, in the main part, due to the rash present in cases of encephalitis due to VZV. Whilst rashes are known to be associated with a range of viral infections including enterovirus, Epstein Barr virus, flavivirus group, influenza and adenoviruses, the majority of rashes in this cohort occurred in cases of VZV encephalitis. However, in our cohort not all cases of VZV encephalitis had a rash present.

**We hope that addresses the questions and comments raised by the Editor and Reviewers and hope these findings will be of interested to the wide audience of your journal.**

**Kind regards**

**Sylviane Defres on behalf of the ENCEPH UK study group**
