# Supplementary material for: Clinical predictors of encephalitis in UK adults–A multi-centre prospective observational cohort study
Source: PLoS One. 2023 Aug 23;18(8):e0282645. doi: 10.1371/journal.pone.0282645 (PMC10446234; doi:10.1371/journal.pone.0282645)
Supplement: Attachment — Submitted filename: Response to reviewers nov22.docx [file pone.0282645.s005.docx]

Staff Editor	Liverpool Brain Infection Group

PLOS ONE	Institute of Infection Veterinary & Ecological Sciences

Ronald Ross Building

University of Liverpool

Dear Dr Lessels	22/11/2022

Thank you for reviewing our manuscript, and for the helpful suggestions which we agree have made the paper clearer and more detailed. Please find here our responses to the points raised by the editor and reviewer.

**Point 1.** *The authors have addressed most of the issues I raised. Unfortunately, I am not fully satisfied. The authors comment to the reviewer that indeed of 10 VGKC positive patients, only 5 were tested for LGI1 and none were positive. Therefore, it is clearly misleading to label this group as VGKC/LGI1 patients, which implies positivity against LGI1. Referring to PMID:32595134 from Sophia Michaels, Paddy Waters and Sarosh Irani, LGI1/CASPR2 negative VGKC findings are “more often misleading than not". Yet the authors still introduce the term VGKC complex in the introduction and include this group which is – frankly - not appropriate anymore in 2022 even though the data and testing was done several years ago. And the lack of LGI1 positivity in the group tested for it (n=5) is not clearly stated in the manuscript. Unless there is serum for retesting available, I would suggest to include that entire group (n=10) into the possible AE (if they fulfil the criteria) but not in the definite group according to Graus et al consensus paper.*

We agree that the landscape of autoimmune encephalitis has changed a lot over the duration of the study, and definitions that once were applicable at the start no longer are. We agree it is inaccurate to therefore label these 10 VGKC positive patients as proven cases. We are limited by the amount of left-over samples from the initial study. But following our initial submission we have reviewed the antibody tests performed, and can confirm that 2 of these 10 patients had left over samples that we were able to evaluate and 1 of these is positive for LGI-1 in CSF (serum was not available for testing). We apologise for this omission from the manuscript previously. Therefore 5 of the 10 patients were originally tested for LGI-1 at the time of the study and a further 2 from left over samples, thus leaving 3 patients that have not had LGI-1 testing performed. The 9 patients with positive VGKC antibodies, either LGI-1 negative or not tested, do all meet the Graus consensus criteria for probable autoimmune encephalitis, because they had MRI in keeping with autoimmune encephalitis and CSF pleocytosis (as per case definition in table 2). Therefore, these 9 patients have been reclassified as probable autoimmune encephalitis, while the 1 LGI-1 positive patient has been reclassified as confirmed autoimmune encephalitis. The tables have been updated to reflect this in the manuscript.

**Point 2.** *The authors have clarified their testing strategy. Indeed, all patients had NMDAR and GAD (and VGKC) testing and eventually patients were also tested for a panel of newer cell surface antibodies. To make this clear for the readers, I would add the number of patients CSF and serum only tested with core testing and the ones tested with the extended panel (either during clinical routine or during retesting of stored samples). This would make it easier to judge the fraction of idiopathic and possible cases that might contain undetected abs like cASPR2. Also please indicate the panel of paraneoplastic onconeuronal abs that were tested.*

We understand the rationale for asking for the detail of exactly which antibodies were tested for to be able to determine which cases may have undetected antibodies. We have given a breakdown for the autoimmune cases the baseline and supplementary autoantibody testing as an appendix.

Of the 38 autoimmune encephalitis patients, all had NMDA, GAD and VGKC tests performed. Unfortunately we do not have complete details of whether antibodies were tested in serum, CSF or both from the referring centres, as in several cases only the list of tested antibodies was reported. Of the 10 cases with positive VGKC antibody, 5 were also tested for LGI1 by local sites and a further 2 by the study team on left over samples of which 1 was positive for LGI-1 in CSF (as outlined above). In total, additional antibody testing was performed on 35 patients with left over samples. Of these 2 were classified as autoimmune encephalitis at the recruiting site, the remainder were encephalitis of unknown aetiology. We have now indicated this in the manuscript. The panel used was the Euroimmun autoimmune encephalitis mosaic panel which included glutamate receptor (type NMDA), contactin-associated protein 2 (CASPR2), glutamate receptors (type AMPA 1/2), leucne rich glioma-inactivated protein 1 (LGI1), dipeptidyl aminopeptidase like protein 6 (DDPX) and GABA B receptor. Apart from the single patient with positive LGI-1 antibody (as already discussed), no other patients tested positive on this panel.

The standard onconeuronal antibody panel includes Hu(D), Yo, CV2/CRMP5, Ri, Ma1, Ma2, amphiphysin,,Tr, SOX1, Zic4, titin, recoverin, PKCγ & Purkinje cell antibodies, tested by immunohistochemistry and recombinant immunoblot. We have now included these details in the manuscript. Testing may have varied slightly depending on the protocols of the diagnostic laboratory in the recruiting hospital. Small numbers of patients had additional antibodies tested, including glycine, gliadin and anti-basal ganglia antibodies, depending on the presenting syndrome.

**Point 3.** *Minor comment: In figure 1 Panel Autoimmune: Iglon5 instead of IglonS, paraneoplastic (seronegative ) cases.*

This has been rectified.

We hope you will feel that we have now done everything possible to address the various issues raised by the reviewers, especially taking into account the fact that the original work was done some time ago. The paper has important messages about clinical features that distinguish herpes simplex virus encephalitis from autoimmune encephalitis to guide presumptive treatment, and we hope you will agree that it is now ready for publication in your journal.

Kind regards

Sylviane Defres on behalf of the ENCEPH UK study group
